
JOURNAL INFORMATION
==============================
NLM Title Abbreviation: ScientificWorldJournal
Journal ID: ScientificWorldJournal
NLM Journal ID: 101131163
Journal ID: TSWJ

The Scientific World Journal

ISSN: 2356-6140
EISSN: 1537-744X
Publisher: Wiley

ARTICLE INFORMATION
==============================
PMCID: PMC6009366
PMID: 29973803
DOI: 10.1100/tsw.2002.29
Article ID: 368424
Article version: 1
Subjects: Short Report

Subdomains in the F and G Helices of Bacteriorhodopsin Regulate Structural Changes

Martinez Lynell C.
Turner George J. *
Department of Physiology and Biophysics Neurosciences Program University of Miami School of Medicine, Miami, FL, USA
*George J. Turner: gturner@miami.edu
Publication date: 2002
Electronic publication date: 2002 Apr 18
Volume: 2
First page: 57
Last page: 58
Copyright: Copyright © 2002 Lynell C. Martinez and George J. Turner.
Copyright year: 2002
License: This is an open access article distributed under the Creative Commons Attribution License, which permits unrestricted use, distribution, and reproduction in any medium, provided the original work is properly cited.
License URL: https://creativecommons.org/licenses/by/3.0/

Funding: National Science Foundation MCB-9817140 American Heart Association AHA9804168V

==============================
Advances in Gene Technology: The Genome and Beyond –
Structural Biology for Medicine (Proceedings of the 2002 Miami
*Nature Biotechnology* Winter Symposium)
*TheScientificWorld* 2002, 2(S2), 57–58
ISSN 1532-2246; DOI 10.1100/TSW.2002.29

### SUBDOMAINS IN THE F AND G HELICES OF BACTERIORHODOPSIN REGULATE STRUCTURAL CHANGES

Lynell C. Martinez and George J. Turner§
Department of Physiology & Biophysics, University of Miami School of Medicine, Miami, FL 33101
§gturner@miami.edu

**INTRODUCTION**. Bacteriorhodopsin is a small polytopic membrane protein containing seven-transmembrane α-helices coupled to retinal via Schiff base linkage to Lysine 216, near the middle of the seventh helix. Light energy involves photoisomerization of the retinal from all-*trans* to 13-*cis*,15-*anti* followed by transitions between a linear series of intermediate conformations[1].

$BR_{568}$ ⌫ J ⌫ $K_{630}$ ⌫ $L_{550}$ ⌫ $M1_{412}$ ⌫ $M2_{412}$ ⌫ $N_{550}$ ↔⌫ $O_{640}$ ⌫ $BR_{568}$

Coupled structural changes regulate the $pK_a$s of critical proton binding sites leading to proton transport. The structures of the ground state and M2 are known and demonstrate that reorientation of the F and G helices comprise the major structural change[2]. The molecular interactions that drive the transitions between these intermediates are still unknown.

Replacement of aspartate 85 (in helix C) with an asparagine (D85N) isolates a subset of the conformational transitions accessed during proton translocation[3]. In the absence of photoexcitation, three spectrally distinct species exist in equilibrium and their relative concentrations are regulated by pH. At neutral pH, the protein exists in an O-like state ($\lambda_{max}$ = 615 nm, appears blue). As the pH is raised, an N- ($\lambda_{max}$ = 570 nm, appears purple) and M-like state ($\lambda_{max}$, 410 nm, appears yellow) become populated. The transitions between M, N, and O reset the major structural change and proton binding site $pK_a$'s.

**METHODS.** To investigate the molecular interactions driving the large-scale conformational changes of the bacteriorhodopsin photocycle, we used *in vitro* gene assembly to perform a cysteine scan of the F and G helices of D85N. Cysteine mutations that disrupt amino acid interactions critical to the transitions between the M, N, and O intermediates will perturb the pH dependent spectral changes of D85N.

We developed a whole cell reflectance spectroscopic screen to evaluate the intermediate state transitions of the 59 mutants generated during a scan. Cell suspensions were transferred to a nylon membrane and equilibrated at pH 7.5, 9.0 and 10.5. Whole cell spectra were recorded at the reflectance port of an RSA 150 integration sphere coupled to a Perkin-Elmer λ18 UV/Vis spectrophotometer. Mutations with similar spectral phenotypes were mapped to the amino acid positions in the 3-Dl structure of BR (1C3W, [4]).

**RESULTS AND DISCUSSION**. Three discrete clusters of amino acids affect the accumulation of M, N and O, indicating that intermediate state structures and the interactions regulating their linking transitions are distinct. The cluster distributions suggests that: (1) the extramembranous loop region is involved in the accumulation of N and M, (2) lipid-protein interactions regulate the formation of N (Fig. 1) and (3) the amino acid side chain interactions in the extracellular portion of the interface between helices G and A regulate the accumulation of the M. The amino acids involved in the transitions are remote from the location of the major structural change indicating that long range energetic coupling regulates the movements of transbilayer helices.

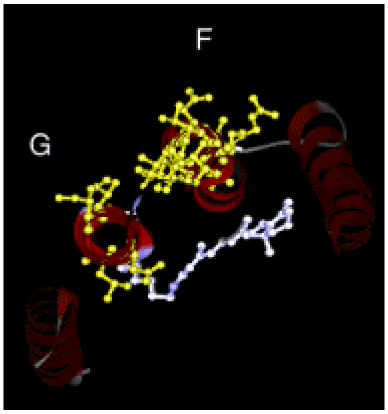

**FIGURE 1**. Structure function map of mutants that affect accumulation of N. View is from the extracellular side orthogonal to the membrane plane.

**ACKNOWLEDGEMENTS**. Support is acknowledged in the form of grants from the National Science Foundation (MCB-9817140) and American Heart Association Fellowship (AHA9804168V) to LCM.

**REFERENCES**

1. Lozier, R.H., Bogomolni, R.A., and Stoeckenius, W. (1975) Biophys. J. 15, 955–962.
2. Subramaniam, S. and Henderson, R. (2000) Biochim. Biophys. Acta 1460, 157–165.
3. Turner, G.J., Miercke, L., Thorgeirsson, T., Kliger, D., Betlach, M.C., and Stroud, R.M. (1993) Biochemistry 32, 1332–1337.
4. Luecke, H., Schobert, B., Richter, H.T., Cartailler, J.P., and Lanyi, J.K. (1999) Science 286, 255–261.
